# Implementing Mental Health Promotion Initiatives—Process Evaluation of the ABCs of Mental Health in Denmark

**DOI:** 10.3390/ijerph17165819

**Published:** 2020-08-11

**Authors:** Carsten Hinrichsen, Vibeke Jenny Koushede, Katrine Rich Madsen, Line Nielsen, Nanna Gram Ahlmark, Ziggi Ivan Santini, Charlotte Meilstrup

**Affiliations:** 1 The Danish National Institute of Public Health, University of Southern Denmark, Studiestraede 6, 1455 Copenhagen, Denmark; krma@sdu.dk (K.R.M.); linn@sdu.dk (L.N.); naah@sdu.dk (N.G.A.); zisa@sdu.dk (Z.I.S.); chme@sdu.dk (C.M.); 2 Department of Psychology, University of Copenhagen, Oester Farimagsgade 2A, 1353 Copenhagen, Denmark; vjk@psy.ku.dk

**Keywords:** mental health promotion, well-being, salutogenesis, implementation, partnership, process evaluation, mixed methods

## Abstract

Treatment and prevention alone are unlikely to make a significant difference in reducing the burden of poor mental health and mental illness. Therefore, mental health promotion (MHP) initiatives are advocated. In 2014, the ABCs of mental health (ABCs) partnership was established in Denmark; in the partnership, partner organisations, e.g., municipalities and NGOs, use a research-based framework for MHP, the ABC-framework, to develop and implement MHP initiatives. This paper has two aims: (1) to outline the overall characteristics of these MHP initiatives; and (2) to explore local coordinator and stakeholder perceptions of the implementation processes and the impact of the MHP initiatives. Questionnaire surveys, individual interviews and group interviews were conducted during 2017–2020. The MHP initiatives were grouped according to three strategies: building MHP capacity, campaign activities to promote mental health awareness and knowledge and establishing and promoting opportunities to engage in mentally healthy activities. The ABC-framework was positively received and viewed as providing relevant knowledge for working with MHP as well as fostering intersectoral and interprofessional collaborations. However, using a bottom-up approach to develop and implement MHP initiatives can be time-consuming and resource demanding, and it requires a deliberate balancing of local adaptability and concrete guidance when engaging stakeholders and implementers. Overall, using the ABC-framework to develop and implement MHP initiatives holds great promise for advancing and promoting MHP practice.

## 1. Background

Mental health problems constitute one of the major global burdens of disease with wide ranging negative consequences at the individual, community and societal level [1,2,3,4,5]. Within the World Health Organization (WHO) and The European Commission and among international researchers from the fields of public health, psychology and psychiatry, there is a growing recognition that targeted prevention and treatment alone are unlikely to make a significant difference in reducing the burden of disease caused by poor mental health. They argue that there is a need for promoting public mental health through effective mental health promoting initiatives [6,7,8].

The WHO defines mental health as “a state of well-being in which the individual realizes his or her own abilities, can cope with the normal stresses of life, can work productively and fruitfully, and is able to make a contribution to his or her community” [4]. Thus, mental health is more than the absence of mental illness and comprises terms such as well-being and resilience [6,9]. In this paper, MHP is conceptualised as “any action taken to maximise mental health and well-being among populations and individuals that focuses on improving social, physical and economic environments that affect mental health, and enhancing the coping capacity of communities as well as individuals.” [10]. The focus of MHP is on strengthening and promoting resources and protective factors for mental health, as opposed to prevention where the focus is on reducing risk factors for mental ill health—i.e., ”what can be done to keep people healthy or to become even healthier?”, rather than “what can be done to avoid illness?” [6]. Thus, MHP is relevant to the whole population regardless of age or physical and mental states of health [6,11].

Key determinants of mental health are found in the settings of everyday life [11]. This implies that MHP is not only a concern for professionals within the healthcare sector (e.g., hospitals and general practice) but is relevant for a wide range of professions. MHP initiatives cover activities operating at the individual, organisational and community level, and they can be categorised as universal (addressing the whole population), selected (targeting subgroups) or indicated (targeting individuals) [6,11]. Universal initiatives drawing on intersectoral and interprofessional approaches are highlighted as promising to achieve effective and sustainable MHP initiatives [8,11,12,13]. Intersectoral and interprofessional collaborations should aim for synergistic impacts and outcomes by mobilising, utilising and sustaining resources contributing positively to MHP [12]. In terms of the determinants that should be targeted within MHP, there is general agreement for the need to improve mental health knowledge and awareness in the general population, public sector and private sector and to provide and promote opportunities for individuals to engage in mentally healthy behaviours [6,10,11].

In Denmark, municipalities are accountable by law to initiate health promoting efforts for their citizens [14]. According to the Danish Ministry of Health, this also includes MHP efforts and these should be designed and implemented within local settings in everyday contexts, such as family settings, day care centres, schools and workplaces [15]. However, two national studies on MHP activities in the Danish municipalities, from 2013 and 2015, indicate a lack of MHP capacity, e.g., knowledge and competencies among service-providers, resulting in little action [16,17].

In 2014, the Danish intersectoral partnership of the ABCs of mental health was founded [9]. The overall aim of the partnership is to promote public mental health in Denmark by bridging the gap between international recommendations, research, policy and practice related to MHP. The partner organisations develop and initiate MHP initiatives within their local settings based on a research-based framework for understanding and working with MHP: the ABC-framework. The ABC-framework was designed to not only reduce the complexity surrounding the concept of mental health for the population at large but also to provide service-providers, health professionals and volunteers with a practical framework for practicing MHP.

The aim of this paper is two-fold. First, we outline the overall characteristics of the MHP initiatives that are based on the ABC-framework and developed and implemented by partner organisations of the ABCs partnership. Secondly, we explore local coordinator and stakeholder perceptions of the implementation processes and the impact of these MHP initiatives. The findings of this paper add to the scarce practice-oriented and research-based literature on translating widely advocated recommendations of applying universal, intersectoral and interprofessional strategies for MHP.

### 1.1. The ABCs of Mental Health

#### 1.1.1. Origin of the ABCs of Mental Health

The ABCs in Denmark is inspired by the Australian MHP campaign Act–Belong–Commit which was adapted to a Danish context by researchers at the National Institute of Public Health (NIPH), University of Southern Denmark [9]. The ABCs is based on the idea of promoting mental health through guidance on what makes people mentally healthy, similar to health promotion and prevention efforts related to other health topics, e.g., alcohol, tobacco and physical activity [10]. This information is compiled and communicated as a feasible and actionable framework for MHP practice: the ABC-framework. The framework is centred around three domains Act, Belong and Commit:

“‘Act’ means that individuals should strive to keep themselves physically, socially and cognitively active. […]

‘Belong’ refers to being a member of a group or organisation (whether face-to-face or not), such that an individual’s connectedness with the community and sense of identity are strengthened. […]

‘Commit’ refers to the extent to which an individual becomes involved with (or commits to) some activity or organisation. Commitment provides a sense of purpose and meaning in people’s lives.”

[10]

The three domains are derived from primary research on lay peoples understandings of mental health promoting behaviour and reviewing the scientific literature [10]. Recent longitudinal population-based studies have demonstrated the mental health promoting effects of the factors pertaining to these domains have [18,19,20].

The ABC-framework builds on a salutogenic rather than a pathogenic orientation and is therefore in line with the aforementioned definition of MHP. In short, the focus is on factors that support human health and well-being rather than on factors that cause disease (for more on salutogenisis, see [21]). In the process of adapting the campaign to the Danish context, the three domains were transformed into a slogan of three action-oriented messages: *do something active, do something with someone, do something meaningful* (in Danish: “*Gør noget aktivt, gør noget sammen, gør noget meningsfuldt*”) [22].

#### 1.1.2. Organisation of the ABCs of Mental Health

**Figure 1:**
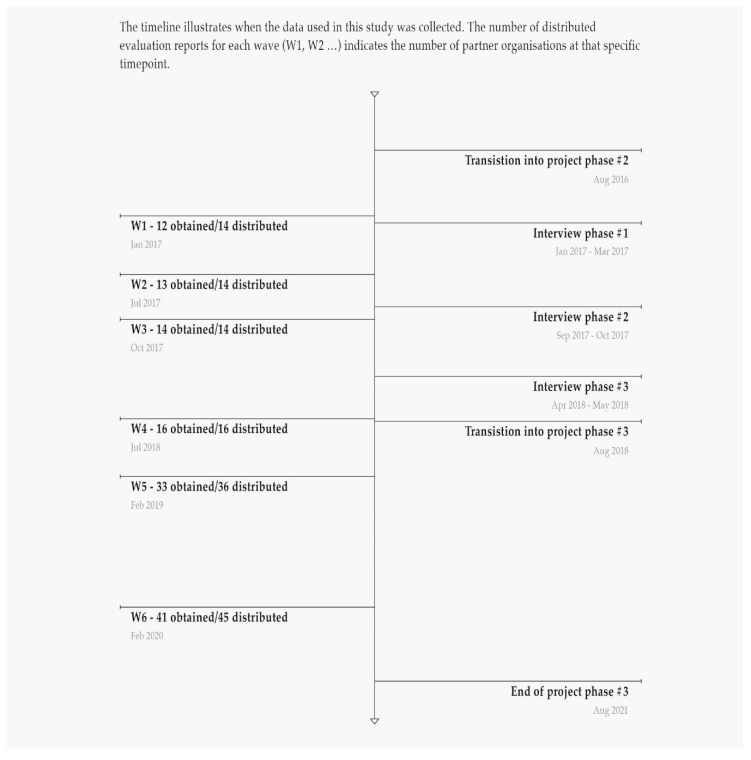
Date collection timeline.

Starting with five partner organisations in 2014, the ABCs partnership has continuously expanded and currently counts more than 46 partner organisations. Further details on partnership expansion are shown in Figure 1. Partner organisations are both public and private organisations; at the time of the latest data collection, there are 25 municipalities, 11 NGOs, 4 unions and 6 other types of organisations.

Based on a bottom-up, community development approach, most partner organisations develop and implement MHP initiatives aiming “*to influence individual behaviour and create supportive environments for fostering and maintaining mental health*” [9]. The bottom-up approach implies that the partner organisations are not provided with exact implementation protocols or standardised manuals. To support this work, some partner organisations receive funding from the ABCs partnership to contribute with specific services to the partnership [22]. Researchers at the NIPH lead and coordinate the partnership and conduct research relevant to the work with MHP in the partnership including a formative process evaluation. The Danish Mental Health Foundation (DMHF) (*Psykiatrifonden*) is responsible for developing and coordinating campaign activities. In addition, the DMHF provides support for adapting campaign materials to local settings and offers communication workshops. The Centre of Prevention in Praxis (CPP) (*Center for Forebyggelse i Praksis*), under KL-Local Government Denmark, provides implementation support and counselling to all municipalities in the partnership. The Healthy Cities Network (*Sund By Netværket*) ensures dissemination of knowledge to municipalities in their network. Together, the Danish Sports Association (DGI) (*Danske Gymnastik-og Idrætsforeninger*), the Danish Scouts Association (*Det Danske Spejderkorps*), the NIPH and the CPP provide training for frontline personnel and volunteers in MHP and the translation of the ABC-framework into local MHP initiatives.

Each partner organisation has at least one local coordinator (ABC-coordinator). Besides coordinating the development and implementation of ABC-initiatives at a local level, the ABC-coordinators also act as links between the partnership and their own organisations. For the purpose of networking and knowledge exchange, the ABC-coordinators are invited to partnership network meetings four times a year and to use an online platform for internal file sharing, accessible to all ABC partners. Coordinated by the NIPH, the form and content of the network meetings are based on the needs and requests expressed by the ABC-coordinators. The network meetings are also used for presenting and discussing findings of the process evaluation to stimulate and facilitate further development of the partnership and MHP initiatives initiated by the partner organisations.

The ABCs partnership has been granted funding for three project phases: 2014–2016, 2016–2018 and 2018–2021. Further information on the background and organisation of the ABCs can be found elsewhere [9,10,18,19,20,22,23].

## 2. Methods

### 2.1. Study Design

This paper draws on mixed method data from the formative process evaluation of the ABCs. The aim of the process evaluation is to document and guide the implementation of local MHP initiatives among partner organisations. The process evaluation was initiated in 2016 and is ongoing. Study participants are ABC-coordinators and their superiors from the partner organisations. Data were collected through evaluation questionnaires consisting of a qualitative and quantitative part, semi-structured individual interviews and semi-structured group interviews. Data were collected at multiple time points from January 2017 to February 2020. A data collection timeline is presented in Figure 1.

### 2.2. Ethics

All participants were informed orally and in writing about the purpose of the process evaluation, that participation was voluntary and that they had the possibility to withdraw their statements at any time before the publication of the results. Written consent was obtained from all interviewees. The formative process evaluation received institutional ethical approval from the University of Southern Denmark (The formative process evaluation received institutional ethical approval from the University of Southern Denmark, No. 10.621, date of approval Dec 1st 2015 (renewed Aug 15th 2019)) and conducted in compliance with The General Data Protection Regulation and the principles of the Helsinki Declaration [24].

### 2.3. Evaluation Questionnaires

Over six waves (W1–W6), 141 questionnaires were sent out and 128 returned (response rate = 91%) (see details in Figure 1). At each wave, the local ABC-coordinators were asked to complete one questionnaire, possibly in collaboration with key stakeholders (e.g., from higher management levels). The questionnaires covered the following topics: motivation for joining the network and history of participation; local organisation and dissemination; collaboration with other partners and local collaborations; development of new materials and activities; facilitators and positive experiences using the ABC-framework; obstacles and barriers; and recommendations to other partners.

The latest questionnaire, W6, consisted of additional items measuring the perceived impact of working with the ABCs. These items, 14 in total, were developed and tested by researchers at the NIPH for the purpose of evaluating the ABCs (Table 1). Of all items, 13 were statements which the respondents were asked to rate on a five-point Likert scale. Five of these items pertained to the individual level and eight items to the organisational level. Further, a categorical item was used to assess perceived impact at the individual level.

### 2.4. Interviews

Five semi-structured individual interviews and 12 semi-structured group interviews were conducted. Interviewees were local ABC-coordinators (consultants and one student assistant) and their superiors (municipal heads of department, secretariat director and secretary-general) from two municipalities, one NGO and one union. These four organisations were purposefully chosen [25] with the aim of including organisations working with the ABC-framework in diverse ways. Interviews were conducted at the participants’ workplaces at three time points (see Figure 1). In total, 15 individuals were interviewed. Most of the interviewees were re-interviewed once or twice. However, due to staff turnover, some interviewees were only interviewed once. Through the interviews, we gathered in depth information on participants’ perceptions on the following topics: the organisation’s role in the partnership; motivation and history of participation; local organisation and dissemination; experiences with local ABC-initiatives; planned ABC-initiatives; campaign congruence with existing objectives and activities; satisfaction with being part of the partnership; and creating sustainable MHP initiatives. The interview data supplemented the questionnaires by generating more in-depth information on participants’ experiences and perceptions.

The interviews were audio recorded. A summary of each interview was written using a literary style [26] with the purpose of condensing the data material but holding on to the overall meaning of the interview content. Based on preliminary findings of the analyses, some passages were transcribed verbatim allowing extraction of passages and citations used for further analyses and reporting the findings. The transcripts and this paper were de-identified, i.e., names of people and places were changed, to prevent participants identity to be revealed.

### 2.5. Analyses

To describe the overall characteristics of the MHP initiatives among partners, data from the questionnaires were thematically analysed [27]. First, MHP initiatives were identified in the data. Secondly, to illustrate and provide an overview of the characteristics of the MHP initiatives under investigation, clusters of MHP initiatives were discussed among co-authors and underwent several iterations. The initiatives were grouped according to three strategies for MHP: (1) building capacity to work with MHP; (2) campaign activities to promote mental health awareness and knowledge; and (3) establishing and promoting opportunities to engage in mentally healthy activities.

To describe local coordinator and stakeholder perceptions of the implementation processes and the impact of the MHP initiatives, a thematic analysis was conducted on the qualitative data. For this purpose, the six steps of conducting template analysis described by Brooks et al. were followed [28]. The Consolidated Framework For Implementation Research (CFIR) [29] was used as a theoretical framework to guide this analysis. The CFIR is a pragmatic meta-theoretical framework, presenting key factors and mechanisms that are of potential importance to implementation processes [29]. The framework can be used to guide formative evaluations and guide post-implementation explorations of implementation processes [29]. The five major themes of the CFIR were applied as a priori themes: characteristics of individuals, characteristics of the intervention, process, inner setting and outer setting [29]. In the analytical process, the authors experimented with clusters of subthemes within each theme to compare, contrast and link the content of each theme. To increase the validity of the qualitative findings, respondent validation [30] was performed by discussing preliminary findings with ABC-coordinators at network meetings. The software NVivo 12 (QSR International) was used for managing the qualitative data and assisting analytical processes.

The coordinator and stakeholder perception of the impact of the ABCs was also analysed through descriptive statistics of the quantitative data from W6. In total, 45 questionnaires were sent out and 41 were returned. One respondent only completed the qualitative part of the questionnaire. Thus, the quantitative results are based on data from 40 respondents (response rate = 89%). Frequencies are reported in Section 3.3. Microsoft Excel was used for the quantitative analysis.

## 3. Findings

The findings are presented in three parts. First, we outline the overall characteristics of the MHP initiatives that are based on the ABC-framework and developed and implemented by ABC partners. Next, we use the five themes of the CFIR to structure the presentation of local coordinator and stakeholder perceptions of the implementation processes and the impact of these MHP initiatives. Finally, we present the findings of the quantitative analyses of the perceived impact.

### 3.1. Overall Characteristics of the MHP Initiatives—Three Strategies

The MHP initiatives developed and implemented by the partners in the ABCs partnership all shared the same overall goal of promoting mental health. They differed on other aspects such as short term aims, i.e., targeted determinants of mental health and the level of intervention (e.g., on an individual, organisational and community level) and the target population (e.g., end-users, i.e., the persons who’s mental health is targeted and service providers). The initiatives were grouped according to three different strategies:

1. building capacity to work with MHP (e.g., by providing staff training and promoting intersectoral and interprofessional collaboration);
2. campaign activities to promote mental health awareness and knowledge (e.g., online advertisements and campaign events); and
3. establishing and promoting opportunities to engage in mentally healthy activities (e.g., volunteer led walking groups and community kitchens).

The division between the three strategies is not clear-cut and they sometimes overlap because the initiatives build upon each other. For example, capacity building efforts at an organisational level were often followed by campaign activities and establishing and promoting opportunities for end-users to engage in mentally healthy activities. However, the categorisation into three strategies is a means of illustrating and providing an overview of the characteristics of the MHP initiatives under investigation.

#### 3.1.1. Building MHP Capacity

**Figure 2:**
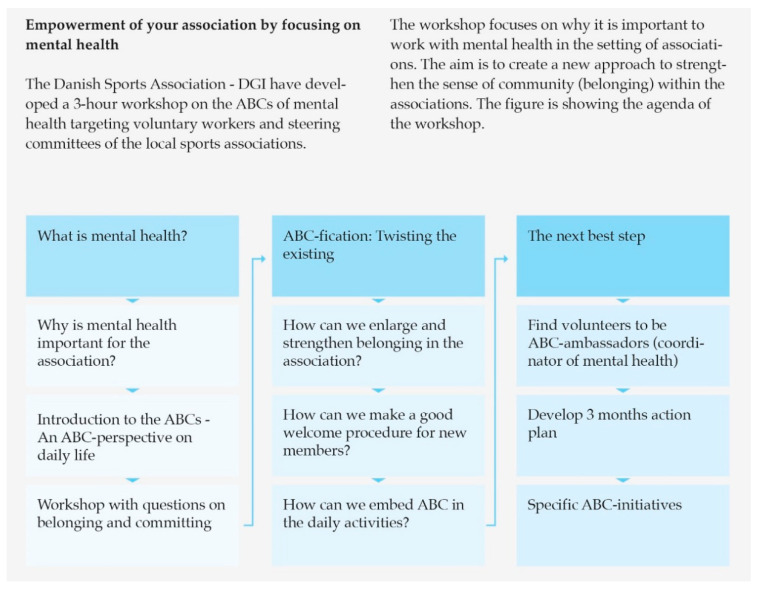
Example of capacity building activity.

Building MHP capacity constituted a major part of the partners’ activities. The capacity building efforts generally aimed to increase knowledge about MHP and to improve relevant organisational structures, e.g., through the training of employees and volunteers and creating local and national intersectoral and interprofessional collaborations. Participants viewed building MHP capacity as a step towards improving and promoting MHP practices, e.g., by enabling and encouraging service providers and volunteers to create and promote mentally healthy activities and environments. Training programs targeting different groups (e.g., employees in municipalities and volunteers in NGOs) were developed, tested and refined by single partner organisations or in collaboration between several partner organisations including the NIPH. The training programs were primarily designed as one-day workshops focusing on how mental health and MHP is conceptualised within the ABC-framework; how the framework can be translated into locally adapted MHP practices and activities; and health communication skills. Figure 2 presents an example of a workshop focusing on capacity building. The partnership network meetings and an online platform for internal file sharing, accessible for all ABC partners, were used for sharing knowledge, e.g., materials and exercises for training purposes and experiences working with the ABC-framework.

#### 3.1.2. Campaign Activities

**Figure 3:**
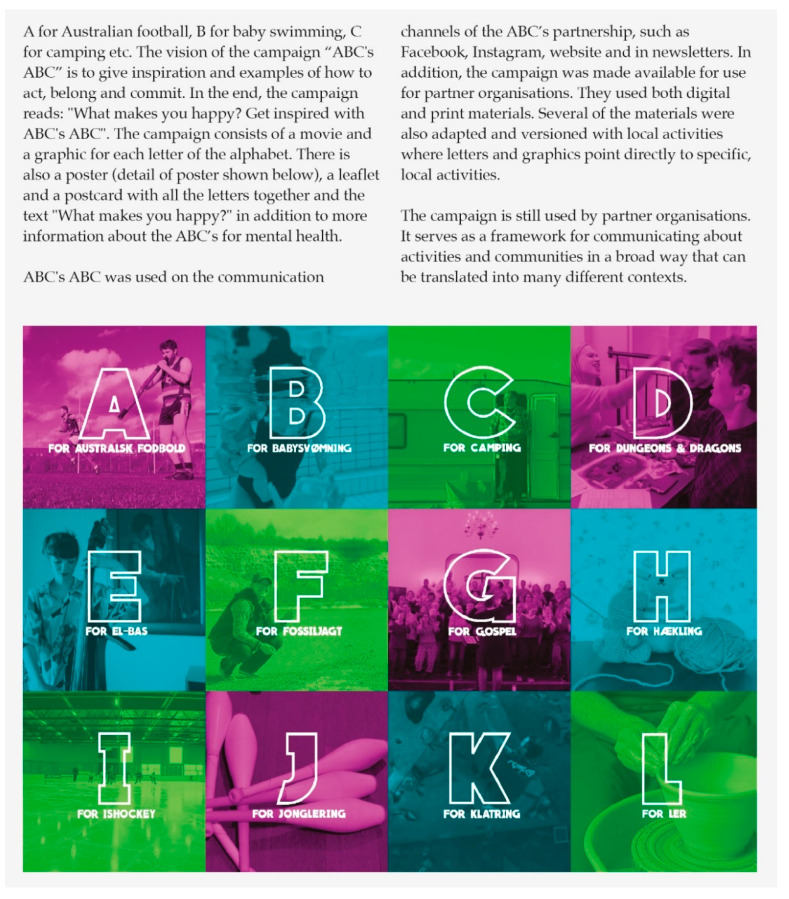
Example of campaign activity: ABC’s ABC.

The second group of MHP initiatives represent various forms of campaign activities developed and/or adopted and executed by the partner organisations. These aimed at increasing mental health awareness and knowledge about how to sustain and promote the mental health of oneself and others. The three domains of the ABC-framework were used as a foundation for developing the campaign activities. In line with the salutogenic approach of the ABC-framework, the campaign activities applied positive appeals, e.g., highlighting the benefits of specific behaviours such as taking up an old hobby using a humoristic approach. Campaign materials were developed by individual partner organisations or in collaboration within the partnership and supported by the DMHF. Figure 3 presents an example of a campaign activity. Campaign materials included print and video advertisements, information leaflets, games and merchandise such as T-shirts, paper fortunes, conversation guides and umbrellas. The channels and settings of communication used for the campaign varied, e.g., social media, newspapers, webpages of the partner organisations, educational institutions and externally initialised events such as music festivals and fitness events. The campaign activities varied in terms of whether they targeted the general population or specific groups, e.g., children in primary schools or adolescents at university colleges.

#### 3.1.3. Mentally Healthy Activities

**Figure 4:**
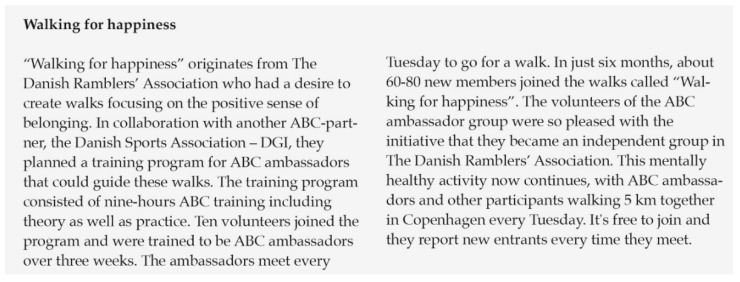
Example of mentally healthy activity.

The MHP initiatives pertaining to the third strategy aimed at establishing and promoting opportunities for end-users to engage in mentally healthy activities. These initiatives were often outcomes of capacity building efforts, as described above. Activities were characterised as being mentally healthy if they were in line with the ABC-messages. For example, several partner organisations arranged community kitchens aimed at engaging and fostering a sense of belonging among community members. In addition, partner organisations used the ABC-framework as a theoretical lens to examine existing activities and services and adjust or refine practices where relevant. This method was labelled “*ABC-fication*” among some partners and was used to boost aspects related to one or more of the three domains within an existing activity or service. For example, several partner organisations increased their efforts in promoting inclusive environments and a sense of belonging among their target groups within existing community activities (for an example, see Figure 4).

An important aspect of several initiatives within this strategy was to promote activities and services by branding or rebranding them as mentally healthy. By highlighting the mental health promoting effects of these activities and services, the branding and rebranding specifically aimed at encouraging target groups to proactively engage in mentally healthy activities. These efforts were similar to the campaign activities but differed in the sense that their primary aim was to increase participation in specific activities and utilisation of specific services, whereas increasing mental health awareness and knowledge was a secondary aim.

### 3.2. Qualitative Findings of the Perceptions of the Implementation Process and the Impact

#### 3.2.1. Characteristics of Individuals

Individuals involved in implementation processes are not passive recipients or mediators; they experiment with, evaluate, seek meaning in and develop feelings (positive or negative) for the innovation or intervention at hand [29]. The first theme from our analysis is concerned with the participants’ (of this study) perception of and beliefs about the ABC-framework.

##### Attitudes toward the ABCs

Overall, the aim and underlying assumptions of the ABCs (i.e., to promote mental health by applying a salutogenic approach and collaborating across sectors, professions and organisations) generally invoked positive reactions from ABC-coordinators, decision makers and potential implementers from various sectors and organisations. They described the ABC-framework as useful in terms of facilitating simple, accessible and tangible communication about MHP with decision makers, service providers and volunteers. Some participants substantiated this by stressing that the plain and simple wording of the ABC-messages made the framework relatable to various groups of people and helped to break down the otherwise complex concepts of mental health, MHP and health promotion. Participants also praised the broad applicability and adaptability of the ABC-framework as this was considered to promote MHP practice across organisational levels.

*“So thanks to your three messages [ABC-messages] one is actually enabled in working with the entire health promotion methodology. That is without having to complicate things or talk a lot about it.”*

(Interview, coordinator, municipality 1)

*“The broad frame of understanding encompassed by the ABCs enables working with mental health at all levels; from political decision makers and cross disciplinary management areas down to the local citizen. Furthermore, we can see a great potential in translating the ABC-framework to locally based methods, initiatives and tools to support activities in promoting mental health.”*

(Questionnaire W2, municipality 3)

In line with the above quotation, the ABC-framework proved to be applicable and suitable in various settings such as kindergartens, elementary schools, nursing homes, sports clubs, evening schools, etc. On the one hand, some participants found these broad possibilities of application inhibiting to the process of developing and initiating MHP initiatives, either because they could not decide where to begin or because their efforts were too extensive related to the resources available. On the other hand, these relatively broad possibilities of application of the ABC-framework were appreciated by participants as they viewed the framework as a conceptual basis for linking ongoing and new initiatives across different departments, sectors and/or organisations. According to some participants, the implementation of ABC-initiatives and a subsequent increased attention on MHP resulted in increased staff motivation to collaborate across professional and organisational boundaries. In a questionnaire from a municipality, for example, working with MHP and the ABC-framework is described as having highlighted that several departments within the municipality work with tasks that reach across internal organisational boundaries, have health promoting effects and would benefit from input from other departments. Therefore, the dissemination of the ABC-framework and the implementation of ABC-initiatives were perceived as promoting synergistic effects through integrated, intersectoral and interprofessional MHP efforts.

#### 3.2.2. Characteristics of the Intervention

Implementation effectiveness is largely influenced by stakeholders’ and implementers’ perception of the intervention characteristics [29]. In our analysis, participants’ perceptions of the appeal forms of the ABCs were particularly prominent.

##### Credibility and Campaign Appeal Forms

The ABC-framework is research based and the partnership is coordinated by a research institute. This was highlighted by several participants as a strength and as creating credibility and legitimacy. In addition, this was seen as supporting the belief that ABC-initiatives and behaviours in line with the ABC-messages will produce the intended outcomes.

*“The fact that the ABCs of mental health is research-based has created credibility from the beginning—albeit that the three domains [act, belong and commit] are obvious for many, it is still meaningful for people to be confirmed that what they are doing is in the right direction.”*

(Questionnaire W4, NGO 1)

Several participants described the positive appeal forms used in, for example, the campaign materials, as desirable, a new way of thinking about health promotion, and, especially, in non-health-oriented settings, making the campaign materials more attractive and appropriate than if they drew on appeals such as risk and fear.

*“Rather than being completely submerged in risk, treatment, shouldn’t and must, then try looking at the fact that there is something that may benefit you in other areas. To me that is exactly what the ABCs enables. As you say, patient associations entail a sort of community with mutual interests and so on, so to me it (the ABC’s) makes sense in several areas.”*

(Group interview, coordinator, municipality 2)

*“But no one wants to join the scouts because they’re overweight. That’s also not why you take up football or something else. So this ‘hey, you need to find out what makes you happy’ and ‘what it is that gives you the energy to smile at the other cyclists’ and stuff like that, that is the focus that decides if you join the scouts …”*

(Interview, coordinator, NGO 2)

Participants had mixed opinions about the appeal of the Danish name ABC for mental sundhed (the ABCs of mental health) and in which situations it was appropriate to use when communicating to end-users. Some participants preferred not to use the name as they saw it as a disruptive element that might complicate the interaction with specific groups, e.g., older adults and children. Therefore, in some situations, the name was not used explicitly; instead, mental health was talked about in broader terms.

#### 3.2.3. Process

The implementation of new practices, ideas and initiatives may be seen as series of interrelated sub-processes that affect the implementation effectiveness [29]. Our analysis showed that the processes of engaging others, e.g., health promotion planners, service providers and volunteers, were substantial in the process of developing and implementing ABC-initiatives.

##### Engaging Others as an Essential and Challenging Aspect of the Implementation Process

Engaging health and non-health-oriented professionals as implementers and mediators of campaign messages was an essential and relatively time-consuming aspect of the local implementation process. Participants reported that local implementers of the ABC-framework, such as health promotion planners, service providers and volunteers, generally deemed the ABC-framework a relevant and useful tool. The implementers found the framework to be in line with existing health promotion initiatives and with general organisational goals of promoting mental health.

*“Overall, the ABCs of mental health have contributed with a method to talk about mental health and wellbeing as well as incorporating mental health in the health promoting work, because all groups of employees understand the ABC messages because they are simple and relevant for most people.”*

(Questionnaire W4, municipality 1)

*“There is a great interest in and accept of the ABC’s as a method. Most employees with contact to citizens think the ABC’s is meaningful as a framework to talk about being active and sense of community.”*

(Questionnaire W5, Municipality 4)

*“We have been able to concretise and put some simple words on how we can help promote population mental health. Something we probably already did sporadically and in a more complicated manner but that we can now clearly formulate.”*

(Questionnaire W4, NGO3)

However, according to the participants, some practitioners (e.g., pedagogues and health care providers) who were introduced to the ABC-framework deemed it as irrelevant and not contributing with anything new. A common initial attitude and response among practitioners was “*we’re already doing it?*”. Some participants reported it as challenging to attract, involve and/or create commitment among colleagues and external collaborators. This challenge was in some cases linked to the difficulty and complexity of conveying the novelty and relevance of the ABC-framework or how it could be applied in practice, precisely because it was in line with already on-going efforts. In an evaluation report, for example, it is reported: *“There is a little ’The Emperor’s New Clothes” about it—we’re already doing that […]”(Questionnaire W1, municipality 1)*. The challenge of engaging others was also linked to the bottom-up approach and the lack of an exact implementation protocol or standardised manual, i.e., not having a clear picture of what the final MHP initiative should encompass or look like.

*“That is the difficult aspect of this, trying to communicate it (the ABC-framework), concretize it, and make it manageable and actionable. That is those next steps.”*

(Interview, coordinator, NGO 3)

*“… how can we as a municipality motivate and concretize “what’s in it for me” for the local collaborative partners?”.*

(Interview, coordinator, municipality 2)

As a result, a recurring recommendation, from the participants in the initial questionnaires and interviews, was to provide practice-oriented examples of ABC-initiatives and manuals or guides on how to translate the theoretical framework into practice, thereby making the ABC-framework less “*fluffy*”. The challenges related to engaging others left some participants with a feeling of progressing more slowly than expected. However, over time, participants developed several strategies to increase the success of engaging relevant stakeholders. Participants found it effective to present the ABC-framework and potential ABC-initiatives as a means of reaching the goals embedded in the core tasks of the audience. This was viewed as particularly relevant in the context of engaging non-health-oriented professionals and volunteers (e.g., pedagogues, teachers and NGO volunteers). In these cases, participants found it effective to shift the main focus from a MHP perspective, with explicit health related goals, to the audiences’ specific core tasks e.g., learning outcomes for teachers.

*“The ABCs is in many ways a simple tool, but nevertheless a tool that needs to be contextualized and adapted depending on the audience. Many local stakeholders, service providers, and municipal partners don’t consider themselves “mental health ambassadors” although they know that their activities promote wellbeing. Also, they don’t use the words “mental health” in their everyday activities. As an ABC-coordinator you therefore need to be able to relate to the context of various local stakeholders and use words that in their setting can be translated and equal mental health such as “feeling good” and “being happy”. Sometimes that means letting go of the words “mental health” and “the ABCs of mental health” because when it comes down to it, the local stakeholders decide for themselves how the ABCs are to be used and highlighted in their activities. That doesn’t mean that they don’t do ABC-activities—just that they have adapted the messages, so they work and live in their organisation.”*

(Questionnaire W4, municipality 3)

*“It isn’t something extra (you need to do), it is just something that can help support you in reaching the goals you are meant to. That is what I find resonates with people. It isn’t rocket-science. It is a way of communicating so practitioners can see, that this is a method that can help them achieve their goals. … You find resources in working with health promotion that makes their job easier.”*

(Interview, coordinator, municipality 1)

Using participatory methods instead of one-way dissemination of information and providing specific examples of ABC-initiatives was also described as a good strategy to increase the success of engaging individuals. Further, when adapting communication and materials to different target groups or settings, participants highlighted the need to balance the theoretical and practice-oriented elements according to the audience. For example, one participant stated: *“We need positive stories and knowledge that isn’t too heavy.” (Interview, coordinator, NGO 2),* whilst others wanted a thorough introduction to the research and theory base. In addition, presenting a selection of concrete ABC-initiatives and campaign materials was described as a way of increasing the chances of decision makers, implementers and collaborators deeming local ABC-initiatives as feasible, suitable and relevant. Again, participants highlighted the need for the training and intervention materials to be balanced in terms of, on the one hand, allowing participants themselves to identify how the ABC-framework might be applied in their context and, on the other hand, receiving concrete examples and guidelines.

*“The ABC-framework has provided a clear message but has given loose boundaries in relation to developing and letting the ABCs take shape according to the context and resources. To some degree this has been liberating as it has provided the ABC-coordinator with the freedom to act on and to form the project locally. On the other hand, at times, as an ABC-coordinator, you needed an indicator that allowed you to set the standards for your work and guide you in terms of being on the right track.”*

(Questionnaire W4, municipality 3)

Several participants highlighted the importance of feeling well prepared for the task of disseminating and promoting the implementation of the ABCs within their organisations. Therefore, the partnership became a valuable platform where the ABC-coordinators could: (1) exchange experiences with other coordinators; and (2) obtain communication training (e.g., communication workshops), educational and training resources (e.g., slides, exercises and relevant literature) and guidance on how to trigger reflections on how the ABC-framework can be translated into practice by different target groups (e.g., through implementation workshops).

Throughout the project period, a decrease in the reported difficulties with engaging, for example, decision makers and local implementers was evident.

#### 3.2.4. Inner Setting

Implementation processes are affected by several aspects relating to the inner setting of an organisation including formal and informal organisational structures and implementation climate [29]. Our analysis showed that the implementation of MHP initiatives was challenged in certain organisations due to existing organisational structures and requirements.

##### Challenges Related to Producing an Economic Case for MHP Initiatives

Some participants indicated that the requirements found within public organisations in some respects challenged the initiation of MHP initiatives, such as the ABCs. Specifically, they experienced that management and decision makers such as politicians requested an economic case, e.g., cost benefit analyses, before initiating or continuing the implementation of MHP initiatives. This was described as challenging the implementation process of ABC-initiatives because producing this type of economic case was seen as almost impossible due to several aspects: applying a bottom up approach and not having a standardised implementation protocol or intervention; lack of resources to predict changes in population mental health or the potential economic gains (as a result of initiatives); and the relative short time frame of the project combined with aiming at long-term outcomes and effects. For example, a manager from a municipality stated:

“So it depends a lot on the economy of it all. And I also think that it is really really difficult to prove that we might save money. Because it is in the future. It is difficult to calculate how much money we might save, and this is always required, when we do these types of things. How much will we save in the future, because we are doing this now?”

(Interview, stakeholder, municipality 2)

#### 3.2.5. Outer Setting

Similar to the inner setting, several elements of the outer setting of an organisation can influence implementation processes within an organisation [29]. These elements include target groups (e.g., service users and patients) and the degree to which an organisation is networked and collaborates with external organisations. In our analysis of the outer setting, three subthemes related to the target groups, interorganisational collaborations and the ABCs partnership were evident (partnership functioning will be described in another paper).

##### Positive Feedback from End-Users on ABC-Initiatives and ABC-Messages

Several participants reported that end-users adopted a positive attitude towards ABC-initiatives and the ABC-messages, e.g., when communicated through the interactive campaign materials such as paper fortune tellers and conversation guides. The framing of the initiatives was highlighted as being essential for how they were received by end users. According to a number of participants, some end-users felt that the term “mental health” had negative and stigmatising connotations and was deemed unappealing or unintelligible because it could be construed as something theoretical, academic or disease oriented. As implied in the following quotation, some participants overcame this issue by using the ABC-messages or related terms to conceptualise mental health when interacting with end users.

*“I find it a challenge that it is called mental health. It creates a barrier in our world. If it was “happy” or “thriving”. I mean if you could find other words to describe it, because it becomes somewhat clinical. When I say something with mental health I hurry up and add ‘active’ or ‘belonging’.”*

(Interview, coordinator, NGO 2)

##### Promoting Interorganisational Collaborations through MHP

Most participants reported that the topic of mental health and the promotion of holistic, intersectoral and interprofessional approaches were already agendas within their organisations when joining the ABCs. Several participants described the development and implementation of ABC-initiatives as a welcoming opportunity, and the ABC-framework as a resource to operationalise these agendas. In addition, participants voiced a great interest in collaborating with external organisations on the development and implementation of ABC-initiatives. According to the participants, several collaborative initiatives based on the ABC-framework were established successfully. Even though the collaborations were often described as successful, they were also subject to challenges. The use of dissimilar terminology related to the local implementation of ABC-initiatives across the partnership was by some reported as an obstacle in the process of developing and implementing ABC-initiatives in collaboration with others. A discussion of the terminology within the partnership was suggested as a means of preventing misunderstandings by several participants. This was discussed at several network meetings in the partnership, the conclusion being that the term “the ABCs of mental health” first and foremost covers a research-based framework for mental health promotion as well as the organisational partnership.

##### The Partnership as a Source of Resources

The participants described the partnership as a source of valuable resources for the process of developing and implementing ABC-initiatives. The resources referred to were: staff training, educational and campaign materials (e.g., flyers, posters, PowerPoint slides and exercise descriptions); professional network; and knowledge and consultations from experts and practitioners (e.g., practice experiences with working with MHP in various settings from other partner organisations, scientific knowledge from the involved researchers or advice on planning and executing campaign activities). Some of these resources were brought into the partnership by specific partners and others were created within the partnership by a single partner or in collaboration between multiple partners. The partner organisations that received funding from the partnership played an essential role in developing and distributing these resources.

*“At the same time, we can learn something from the other partners. That is the balance that makes a partnership interesting. That we can influence and contribute with something we are particularly good at. But it is also interesting that we can mirror ourselves in other organisations and the way they do things. Or learn something from how others receive this type of project and how they work with it. I find that is the balance that makes it rewarding.”*

(Interview, stakeholder, NGO 2)

Specifically, the educational and campaign materials produced in the partnership were highlighted in several evaluation reports as essential resources for disseminating the ABC-framework and establishing it as an applied resource and, thus, aiding the process of implementing ABC-initiatives locally. In an evaluation questionnaire, it is stated that these materials tap into a lack of organisational and individual resources for initiating and developing MHP initiatives:

“In the ABCs we inform people that even small adjustments and reprioritizing in your everyday life can help promote your mental health. However, not all citizens, organisations or workplaces have the creativity or energy to devise what these changes might be.”

(Questionnaire W3, municipality 3)

Furthermore; the free counselling and services related to communication activities offered to all partners (e.g., developing and planning campaign activities) proved to be particularly valuable to partners as they were perceived to facilitate the development and implementation process of local ABC-initiatives. Most partner organisations used these opportunities, and numerous partners praised these services in the evaluation questionnaires. For example, one participant described how this made it possible for them to develop and try out new concepts which they otherwise would not have had the resources to develop. The centrally coordinated campaign, based on a social marketing strategy and executed by the partner organisations, in connection with local ABC-initiatives was described as creating positive synergy effects. A participant, for example, mentioned that it was meaningful to promote specific campaign messages when the same messages were echoed in the media.

### 3.3. Perceived Impact–Quantitatively Assessed

#### 3.3.1. Characteristics of Respondents

The characteristics of the respondents at W6 are presented in Table 2. Of the respondents, 23 were from municipalities, 10 from NGOs, 3 from unions and 5 from other types of organisations. Twenty-nine respondents (70%) had been involved with the implementation of the ABCs in their organisations for two years or less. The remaining respondents had been involved since 2015 (2%), 2016 (20%) or 2017 (7%). The majority of the respondents stated *health* (71%) as their primary field of professional interest; around one third stated *volunteer work* (34%); and around one fifth stated, respectively, *culture* (22%), *social services* (22%) and *physical activity* (20%) (multiple answers were allowed).

#### 3.3.2. Response Frequencies for Items on Perceived Impact

**Figure 5:**
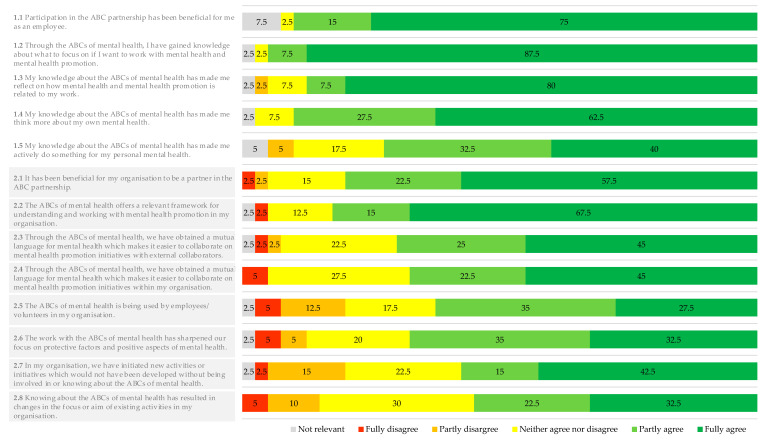
Response frequencies in per cent for survey items on perceived impact.

Response frequencies for items assessing perceived impact using a Likert-scale are presented in Figure 5. Respondents generally agreed that working with the ABCs had an impact on an individual level. For example, when asked whether the participation in the ABCs partnership was beneficial for them as an employee (Item 1.1), 75% of the respondents fully agreed, 15% partly agreed and 2.5% answered neither agreed nor disagreed (7.5% stated not relevant). Further, respondents generally agreed that their knowledge of the ABCs had motivated them do something for their own personal mental health (Item 1.5): 40% fully agreed, 32.5% partly agreed, 17.5% neither agreed nor disagreed and 5% partly disagreed (5% stated not relevant). Overall, between 72.5% and 95% of the respondents partly agreed or fully agreed on the items on the individual level (Items 1.1–1.5).

The diagram is based on data from 40 respondents. Respondents were asked to rate each item. Items 1.1–1.5 pertain to the individual level and Items 2.1–2.9 to the organisational level.

All respondents (i.e., 100%) reported to have talked with colleagues about the ABCs; 82.5% with partners from other organisations; 70% with friends; 77.5% with family; and 12.5% with others (results not shown).

Most of the respondents agreed that working with the ABCs had a positive impact on an organisational level. Overall, between 55% and 82.5% partly or fully agreed on Items 2.1–2.8 pertaining to the organisational level. For example, when asked whether new initiatives or activities had started up as a result of their involvement in or knowledge about the ABCs (Item 2.7), 42.5% of the respondents fully agreed, 15% partly agreed, 22.5% neither agreed nor disagreed, 15% partly disagreed and 2.5% fully disagreed (2.5% stated not relevant). In addition, 67.5% fully agreed and 15% partly agreed that the ABC-framework was relevant for understanding and working with MHP in their organisation (Item 2.2.), while 12.5% neither agreed nor disagreed and 2.5% partly disagreed to this (2.5% stated not relevant).

## 4. Discussion

In this study, we first investigated and described the overall characteristics of the ABC-initiatives that are developed and implemented by the partner organisations of the ABCs. These initiatives were grouped according to three strategies: (1) building MHP capacity; (2) campaign activities to promote mental health awareness and knowledge; and (3) establishing and promoting opportunities to engage in mentally healthy activities. Secondly, we analysed and described local ABC-coordinator and stakeholder perceptions of the implementation processes and the impact of the MHP initiatives. ABC-coordinators and stakeholders deemed the ABC-framework as useful in a range of strategies for MHP and feasible in various settings. Overall, the ABC-initiatives were positively received. However, our results also indicate several challenges to the implementation processes. Participants, particularly from the public sector, reported challenges related to meeting organisational requirements of providing economic cases for investing in MHP initiatives. Other challenges were related to balancing local adaptability and concrete guidance and engaging others in the implementation. The study participants viewed the ABC-framework as providing relevant knowledge for working with MHP and as a means by which to foster intersectoral and interprofessional collaboration on MHP. Overall, these results are in line with evaluation studies on the Act–Belong–Commit campaign in Australia, which, among others, found that the campaign provided a useful framework for teachers to talk about mental health with students [31] and assisting local stakeholders in creating sustainable local collaborations across organisational boundaries [32].

### 4.1. A Novel Approach to MHP?

As our results indicate, the work with the ABC-framework is in some respects similar to strategies and approaches already used across different organisations in Denmark. For example, existing practices in various settings (e.g., schools, libraries and sports clubs) were deemed as being in line with the ABC-framework, and in some cases the ABC-framework was even dismissed by practitioners with the statement “*we’re already doing that*”. This may be explained by the fact that the ABC-framework and the underlying approach builds on existing knowledge from several disciplines [9,10] and, therefore, isolated elements of it may be similar to existing practices. However, the ABC-framework and its underlying approach was conceived as a new approach to practising MHP that complies with the recommendations for MHP [6,8,11,13]. The intended novelty lies in providing a salutogenic oriented conceptual framework for working with MHP based on concrete and actionable guidance as well as linking MHP strategies across different departments, sectors and/or organisations, thus aiming for synergistic effects. The ABC-framework can aid practitioners from various professions and settings to employ a health promoting practice that is based on deliberate decisions regarding which elements of their practice are health promoting—not to be mistaken for more preventive-oriented measures. For most participating organisations, this re-orientation of practice was a desired goal. Through staff training, this goal was pursued by enabling practitioners: (1) to identify these mental health promoting elements fitting their daily practice; and (2) to adjust, if already existing, or to apply them if deemed necessary. Our findings support that the ABC-framework is a relevant contribution for advancing mental health promoting practices, with participants predominantly agreeing that the ABC-framework was relevant for understanding and working with MHP in their organisation. Overall, the findings indicate that developing and implementing MHP initiatives based on the ABC-framework can be characterised as a new approach that builds on existing knowledge.

### 4.2. Balancing Adaptability and Guidance

Initially, a main focus was to provide partner organisations of the ABCs with a research-based tangible framework for developing and implementing MHP initiatives. Applying this type of bottom-up approach which allows for local adaptation is recommended for MHP and is important as a means to create empowerment and sustainability [6,33]. As anticipated, the bottom-up approach was considered a strength by participants in this study as this allowed for tailoring initiatives to local settings and various professions and, thus, facilitated coherent MHP efforts across organisational boundaries. These findings are in line with previous studies on the implementation of the Act–Belong–Commit campaign [31]. It soon became clear, however, that some implementers and ABC-coordinators needed more guidance and concrete examples of how to use the framework in practice. As time has passed, good examples from practice have become more readily available (e.g., in evaluation reports and training materials) and have been shared across the partnership on a regular basis. Hence, challenges pertaining to this have been markedly reduced. The partnership still aims to facilitate room for creative thinking on how the ABC-framework can be translated into practice locally, before giving too many concrete examples and guidelines. In addition, our results showed that applying a bottom-up approach when planning and implementing MHP initiatives can be time-consuming and resource demanding for local organisations and implementers. Based on these findings, we encourage health promotion planners and practitioners to carefully consider the balance between local adaptability and concrete guidance as a means of facilitating an effective process of developing and implementing MHP initiatives based on local needs and resources. In addition, the potentially time and resource demanding processes pertaining to a bottom-up approach should be planned for.

### 4.3. Responsibility for Ensuring MHP Initiatives

The Danish Ministry of Health advocates that MHP initiatives should be designed and implemented within local settings where people live their daily lives such as family settings, day care centres, schools and workplaces [15]. To achieve this focus on MHP in these settings, there is a need for re-orienting policies and practices through a broad engagement across sectors and professions [11]. The emphasis on including non-health-oriented settings might be construed as a placement of responsibility for ensuring the provision of MHP initiatives on the staff working in these settings. However, our findings show that some non-health-oriented professionals and volunteers do not consider themselves as *mental health ambassadors*. This might indicate that they view issues related to MHP as outside their sphere of interest, responsibility and/or expertise, which has been shown in other studies, for example, among teachers and health professionals [34,35,36]. These potentially diverging perspectives between policies and staff and a lack of interest and motivation among implementers may challenge the implementation of MHP initiatives [33] and, overall, the re-orientation of public services towards a practice with a greater awareness of mental health. Such challenges may be amplified when MHP is conceptualised and approached differently across professions and sectors [37,38], which can leave frontline staff with a feeling of mental health as difficult to define and operationalise [34]. This relates to the importance of bringing attention to the process of engaging relevant individuals in implementation, among others, by promoting a mutual understanding of MHP. This is also highlighted by the participants of this study as well as in the literature on implementation and MHP [29,39]. In the case of promoting the implementation of MHP initiatives, this study shows that it can be particularly effective to present MHP initiatives as a means of supporting non-health-oriented professionals and volunteers in conducting their core tasks.

### 4.4. Methodological Considerations

In this paper, we present findings of a process evaluation of a large national partnership outlining how 45 organisations approached the development and implementation of MHP initiatives. We also explore the implementation processes and the impact of the MHP initiatives. These findings add knowledge to the existing research on MHP in practice and are relevant for planning and implementing such initiatives. Findings are derived at using a mixed methods design with data regarding more than three and a half years of partnership work collected at multiple time points (six waves of evaluation questionnaires and three interview phases). This allowed for an iterative exploration over time of development and implementation processes and the impact of MHP initiatives. The analysis was guided by the CFIR [29], which allowed for an exploration of essential factors on multiple levels.

Interviews were only conducted with coordinators and stakeholders from four of 45 organisations, and their perspectives might not be representative for coordinators and stakeholders from other partner organisations. However, the questionnaire data allowed for investigating the prevalence of certain perspectives and preliminary findings among coordinators and stakeholders from a wider segment of partner organisations. Further, preliminary findings were discussed at network meetings within the ABCs partnership, providing the authors with valuable insights for interpreting the data in this study and, thus, strengthening the credibility of the findings. The methodology used in this study was limited, as it is not clear who were involved in the completion of the evaluation questionnaires, as the local coordinators could do so in collaboration with key stakeholders from their organisation. In addition, frontline staff and end-user perspectives are relevant to study in order to better understand implementation processes and impacts [29].

This process evaluation can be characterised as internal, or not independently conducted, as it was conducted by researchers from the NIPH who also lead and coordinate the partnership. Internal evaluations are subject to advantages and disadvantages [40,41]. In this case, the evaluators’ central role in the partnership due to their managerial tasks might have led to participants holding back criticism, overreporting the number of implementation activities and overrating the impact in order to please or avoid conflict with the evaluators [41]. To minimise this type of information bias, study participants were explicitly urged to share their thoughts and experiences without filter during data collection. The choice of conducting internal evaluation was based on the formative purpose of the evaluation by creating “organisational learning” within the ABCs partnership. According to Volkov, this requires informed evaluators with a thorough knowledge of organisational functioning and dynamics [42], which may also lead to more accurate and context sensitive interpretations [41].

Evaluation studies on the long-term effects applying mental health and economic outcomes are needed to fully understand the potential of approaching MHP as in the Danish ABCs partnership. These types of studies were not deemed relevant at the time of initiating the partnership. This was due to the developmental state of the ABCs partnership and the underlying bottom-up approach which, among others, entailed an uncertainty about which specific outcome measures would be relevant to apply. Based on the findings of this study and other evaluation activities, evaluation studies using measures on mental health outcomes are planned.

### 4.5. Implications for Practice

There is consensus that mental health is more than merely the absence of mental illness [43]. However, to date, the focus in mental health—politically as well as scientifically—has primarily been on treatment or prevention of mental illness [8]. As a result, people may perceive the term mental health to be related to mental illness, or even use the terms interchangeably, and to have stigmatising connotations [34,44], which our findings also indicate. Whilst treatment and prevention of mental illness are very important to ensure, strategies solely focused on mental illness cannot stand alone if the aim is to reduce the amount of people in the population that are affected by mental health problems [6,7,8]. WHO, the EU-commission and experts have stated the need to supplement treatment and preventive strategies with mental health promotion and a focus on the positive aspects of mental health and well-being [6,11,43,45]. Being able to join and engage in various leisure- or work-related activities, having a sense of belonging and commitment to something and someone are vital aspects of mental health for everyone regardless of age, gender, income, physical or mental health problems or disabilities [6,11]. Building on and integrating MHP components in existing programmes and practices in local communities should be prioritised from a MHP point of view [13]. It is therefore important that the public and private sector understand how they may be able to promote mental health and wellbeing through contributing to supporting surroundings and empowering various target groups to engage in mentally healthy activities. Our findings offer not only an outline of three relevant strategies for MHP, including examples of MHP initiatives from practice, but also describes insights from implementation processes that are valuable when developing and implementing MHP components in practice. In addition, our results indicate that using the ABC-framework holds promising potential for promoting MHP efforts and collaborations across sectors and professions. The framework may also be a valuable tool for raising awareness regarding opportunities to act, belong and commit in local communities, verbalising mental health as something more than just the absence of disease and as something that is valuable in itself and should be prioritised.

## 5. Conclusions

The MHP initiatives investigated in this study were grouped according to three strategies: building MHP capacity, campaign activities to promote mental health awareness and knowledge and establishing and promoting opportunities to engage in mentally healthy activities. The ABC-framework has proved useful in various types of MHP initiatives and feasible in various settings. However, using a bottom-up approach to develop and implement MHP initiatives can be time-consuming and resource demanding, and it requires a deliberate balancing of local adaptability and concrete guidance when engaging stakeholders and implementers. We found that it can be particularly effective to engage non-health-oriented implementers by presenting MHP and the ABC-framework as means of supporting them in conducting their core-tasks, which might not be explicitly linked to mental health. The findings presented in this paper offer valuable practice-oriented insights relevant to consider planning and practicing MHP. Overall, the ABC-framework has been positively received by local implementers and stakeholders and viewed as a means by which to foster intersectoral and interprofessional collaboration. Overall, using the ABC-framework to develop and implement MHP initiatives holds great promise for advancing and promoting MHP practice. Rigorous effectiveness studies are needed to determine the long-term effects of these MHP initiatives.

## Figures and Tables

**Table 1:** Survey items to measure perceived impact used at W6.

Item	Variable	Questionnaire Item	Response Category
**Individual level**			
1.1	Overall benefit	Participation in the ABC partnership has been beneficial for me as an employee.	1 = Fully disagree, 2 = Partly disagree, 3 = Neither agree nor disagree, 4 = Partly agree, 5 = Fully agree, 0 = Not relevant
1.2	Knowledge about MHP	Through the ABCs of mental health, I have gained knowledge about what to focus on if I want to work with mental health and mental health promotion.	
1.3	Awareness of relationship between MHP and professional tasks	My knowledge about the ABCs of mental health has made me reflect on how mental health and mental health promotion is related to my work.	
1.4	Increased awareness, personal mental health (mind-set)	My knowledge about the ABCs of mental health has made me think more about my own mental health.	
1.5	Behaviour change, personal mental health	My knowledge about the ABCs of mental health has made me actively do something for my personal mental health.	
1.6	Behaviour change, talking about the ABCs	I have talked about the ABCs of mental health with … (multiple answers allowed)	a) Colleagues, b) Collaborators from other organisations, c) Friends, d) Family, e) Others, f) None
**Organisational level**			
2.1	Overall benefit	It has been beneficial for my organisation to be a partner in the ABC partnership.	1 = Fully disagree, 2 = Partly disagree, 3 = Neither agree nor disagree, 4 = Partly agree, 5 = Fully agree, 0 = Not relevant
2.2	Relevant framework for MHP	The ABCs of mental health offers a relevant framework for understanding and working with mental health promotion in my organisation.	
2.3	Mutual language for external collaboration	Through the ABCs of mental health, we have obtained a mutual language for mental health which makes it easier to collaborate on mental health promotion initiatives with external collaborators.	
2.4	Mutual language for internal collaboration	Through the ABCs of mental health, we have obtained a mutual language for mental health which makes it easier to collaborate on mental health promotion initiatives within my organisation.	
2.5	Usage of ABC-framework by employees/volunteers	The ABCs of mental health is being used by employees/volunteers in my organisation.	
2.6	Increased focus on MHP	The work with the ABCs of mental health has sharpened our focus on protective factors and positive aspects of mental health.	
2.7	Initiation of new initiatives	In my organisation, we have initiated new activities or initiatives which would not have been developed without being involved in or knowing about the ABCs of mental health.	
2.8	Modifications and changes in existing initiatives	Knowing about the ABCs of mental health has resulted in changes in the focus or aim of existing activities in my organisation.	

**Table 2:** Characteristics of the respondents of the questionnaire at W6 collected in February 2020.

Characteristic	Category	N (%)
N		41
Type of organisation	Municipality	23 (56)
	NGO	10 (24)
	Unions	3 (7)
	Other	5 (12)
Year of involvement with the implementation of the ABCs	2015	1 (2)
	2016	8 (20)
	2017	3 (7)
	2018	12 (29)
	2019	17 (41)
Primary field of professional interest ^a^	Health	29 (71)
	Culture	9 (22)
	Senior	7 (17)
	Social service	9 (22)
	Non-profit	3 (7)
	Physical activity	8 (20)
	Volunteer work	14 (34)
	Other	6 (15)

^a^ Several answers allowed pr. respondent.
